# Response of Tree Seedlings to a Combined Treatment of Particulate Matter, Ground-Level Ozone, and Carbon Dioxide: Primary Effects

**DOI:** 10.3390/plants14010006

**Published:** 2024-12-24

**Authors:** Valentinas Černiauskas, Iveta Varnagirytė-Kabašinskienė, Ieva Čėsnienė, Emilis Armoška, Valda Araminienė

**Affiliations:** Institute of Forestry, Lithuanian Research Centre for Agriculture and Forestry, Liepų 1, Girionys, LT-53101 Kaunas, Lithuania; valentinas.cerniauskas@lammc.lt (V.Č.);

**Keywords:** tree seedlings, simulation experiment, urban environment, air pollution, CO_2_

## Abstract

Trees growing in urban areas face increasing stress from atmospheric pollutants, with limited attention given to the early responses of young seedlings. This study aimed to address the knowledge gap regarding the effects of simulated pollutant exposure, specifically particulate matter (PM), elevated ozone (O_3_), and carbon dioxide (CO_2_) concentrations, on young seedlings of five tree species: Scots pine (*Pinus sylvestris* L.); Norway spruce (*Picea abies* (L.) H.Karst.); silver birch (*Betula pendula* Roth); small-leaved lime (*Tilia cordata* Mill.); and Norway maple (*Acer platanoides* L.). The main objectives of this paper were to evaluate the seedling stem growth response and the biochemical response of seedling foliage to pollutant exposure. Four treatments were performed on two- to three-year-old seedlings of the selected tree species: with PM (0.4 g per seedling) under combined O_3_ = 180 ppb + CO_2_ = 650 ppm; without PM under combined O_3_ = 180 ppb + CO_2_ = 650 ppm; with PM (0.4 g per seedling) under combined O_3_ < 40–45 ppb + CO_2_ < 400 ppm; and without PM under combined O_3_ < 40–45 ppb + CO_2_ < 400 ppm. Scots pine and Norway maple showed no changes in growth (stem height and diameter) and biochemical parameters (photosynthetic pigments, total polyphenol content (TPC), total flavonoids content (TFC), and total soluble sugars (TSS)), indicating a neutral response to the combined PM, O_3_, and CO_2_ treatment. The chlorophyll response to PM alone and in combination with elevated O_3_ and CO_2_ exposure varied, with silver birch increasing, Norway maple—neutral to increasing, Scots pine—neutral to decreasing, and Norway spruce and small-leaved lime—decreasing. The TPC indicated stress responses in Scots pine, small-leaved lime, and Norway maple under increased combined O_3_ and CO_2_ and in Norway spruce under single PM treatment. Hence, Scots pine and Norway maple seedlings showed greater resistance to increased PM under combined O_3_ and CO_2_ with minimal change in growth, while silver birch seedlings showed adaptation potential with increasing chlorophyll under simulated pollutant stress.

## 1. Introduction

Rapid urbanization increases the amount of air pollutants in the urban environment, harming human health and the ecosystem [1,2,3,4,5]. Despite substantial declines in air pollution across Europe in recent decades, the challenges persist in urban areas. Pollutants such as nitrogen dioxide (NO_2_), ground-level ozone (O_3_), polycyclic aromatic hydrocarbons (PAHs), and particulate matter (PM) frequently exceed the thresholds [6]. The PM (coarse PM with a diameter of 2.5–10 μm and fine PM with a diameter of <2.5 μm) is a prevalent air pollutant released from vehicle exhaust, rubber tire particles, organic chemicals, metals, and dust from the fossil fuel industry. This pollutant significantly threatens human health, especially in urban areas [7]. In particular, PM_2.5_, smaller than 2.5 μm, is recognized as a significant global health issue and is responsible for 0.8 million premature deaths worldwide [8,9].

As an integral part of urban ecosystems, trees are crucial in reducing air pollution by acting as natural filters. Urban areas are frequently distinguished by altered PM, CO_2_, and O_3_ concentrations, primarily mitigated by vegetation alongside other air pollutants [10,11]. Otherwise, O_3_ and PM still stand out as the foremost air pollutants affecting plants, as highlighted by numerous studies [6,12,13,14,15]. Understanding how trees respond to common urban pollutants is critical to preserving and improving the ecosystem services they provide [5]. Although trees growing in urban areas assimilate CO_2_ [16,17], elevated O_3_ concentrations induce biochemical and physiological changes in plants with inhibition of carbon assimilation by photosynthesis when it penetrates the intercellular spaces through stomata [18,19]. Trees can filter air pollutants [20,21]. Several studies have investigated that trees effectively remove PM, which adheres to the surface of plants, effectively extracting them from the air [22,23,24,25,26,27,28,29]. Plants with large leaf surfaces could reduce urban air pollution [20,30]. Other studies noted that coniferous species can more effectively capture PM than broadleaved species [26].

Over recent decades, increasing attention has been paid to the services trees provide in urban areas for mitigating air pollution in a changing climate [16,31,32,33,34]. Previous studies have examined the role of urban forests in reducing CO_2_, a dominant greenhouse gas [31]. Knowing that CO_2_ enters plants through stomata, previous studies have found species-specific differences, with plant species having higher stomatal densities absorbing PM and gaseous pollutants more effectively [16,35]. The PM deposition on the leaves interrupted stomatal leaf exchanges by lowering the CO_2_ assimilation rate (photosynthesis) and the exchange of water (transpiration) [36,37]. While warmer ambient air accelerates tree growth and enhances carbon sequestration, the impact of urban air pollution, containing specific compounds such as PM_10_, exerts a more significant influence on growth than the climate itself [34]. The response of trees to induced stress is highly dependent on the accumulation of specific biochemical compounds within the plant [38]. Biologically active compounds, including photosynthetic pigments and secondary metabolites, play an essential role in the plant’s health, such as the resistance of trees to unfavorable environmental conditions. These compounds are one of the key determinants in the ability of trees to tolerate and adapt to stressors such as pollution [39].

A complex of atmospheric pollutants acting synergistically is likely to increase the stress of trees growing in urban areas at a young age, and an early response could be detected. Although more attention is usually given to mature trees growing for several years in an open urban environment [40], more scientific documentation of the initial effects on young seedlings is needed. This knowledge gap raises concerns about the increased vulnerability of young seedlings and potential implications for subsequent tree development and resilience in urban environments.

Due to limited information on the reactions of young trees, this study focuses on seedlings, assuming that damage occurring at a young age may impact tree development, growth, and health in later years. Additionally, experiments with young trees allow for quicker identification of specific responses to environmental disturbances, such as pollutants. This paper aimed to investigate the early growth (height and diameter) and biochemical effects of simulated air pollutant exposure, focusing on the elevated combined O_3_ and CO_2_ concentrations on young tree seedlings (*Pinus sylvestris* L., *Picea abies* (L.) H.Karst., *Betula pendula* Roth, *Tilia cordata* Mill., and *Acer platanoides* L.) treated with and without PM. We hypothesized that seedlings of different tree species would show species-specific growth and biochemical responses to simulated exposure to PM alone and the combined effects of elevated O_3_ and CO_2_, with potentially stronger responses to the impact of all three factors.

## 2. Results

### 2.1. Stem Growth Response to Pollutant Exposure

**Figure 1:**
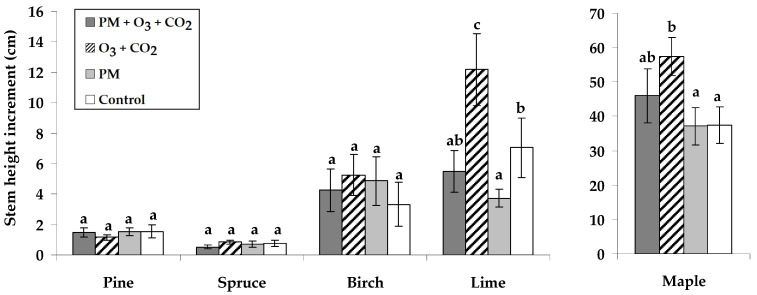
Stem height increments (±SE) of Scots pine, Norway spruce, silver birch, small-leaved lime, and Norway maple seedlings. Experimental treatments: PM + O_3_ + CO_2_ is a treatment with particulate matter (PM) at elevated O_3_ and CO_2_; O_3_ + CO_2_ is the treatment without PM at elevated O_3_ and CO_2_; PM is the treatment with PM at no elevated O_3_ and CO_2_; Control has no PM, no elevated O_3_, and CO_2_ (*n* = 21). Data significance was calculated using the Kruskal–Wallis test followed by Dunn–Bonferroni post hoc test for pairs (*p* ≤ 0.05). Different letters indicate statistically significant differences between treatments within each species.

The growth response included evaluating the changes in increment of Scots pine, Norway spruce, silver birch, small-leaved lime, and Norway maple seedling height and stem diameter at root base under various simulated environmental conditions, including PM, and elevated combined O_3_ and CO_2_ levels. The height increment of each tree species over the experimental period showed that elevated combined O_3_ and CO_2_ generally caused higher height increments across all species, exclusively for small-leaved lime and Norway maple (Figure 1). The stem height of small-leaved lime seedlings decreased due to exposure to PM without elevated O_3_ and CO_2_.

**Figure 2:**
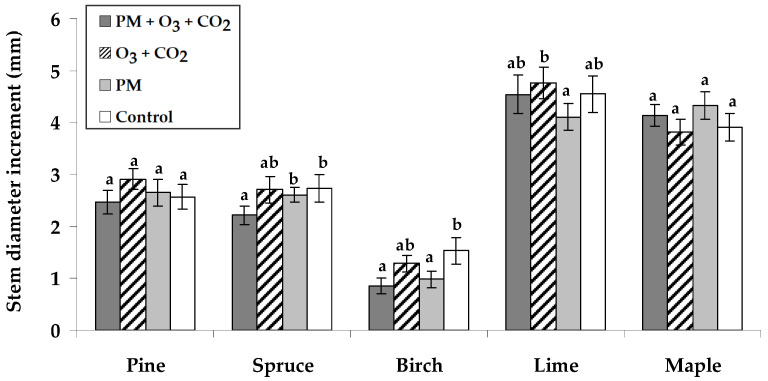
Stem diameter increment (±SE) of Scots pine, Norway spruce, silver birch, small-leaved lime, and Norway maple seedlings. Experimental treatments: PM + O_3_ + CO_2_ is a treatment with particulate matter (PM) at elevated O_3_ and CO_2_; O_3_ + CO_2_ is the treatment without PM at elevated O_3_ and CO_2_; PM is the treatment with PM at no elevated O_3_ and CO_2_; Control has no PM, no elevated O_3_, and CO_2_ (*n* = 21). Data significance was calculated using the Kruskal–Wallis test followed by Dunn–Bonferroni post hoc test for pairs (*p* ≤ 0.05). Different letters indicate statistically significant differences between treatments within each species.

Considering various simulated environmental conditions, the stem diameter increments of seedlings over one vegetation season showed insights into the species-specific responses to these conditions (Figure 2). Compared to the untreated controls, the slightly higher stem diameter increments of Scots pine and small-leaved lime seedlings occurred under elevated combined O_3_ and CO_2_ conditions without PM. The PM exposure with elevated combined O_3_ and CO_2_ resulted in 1.2 times reduced diameter growth for Norway spruce and 1.8 times for silver birch seedlings compared to the untreated controls. The PM without elevated O_3_ and CO_2_ reduced the stem increment of silver birch.

### 2.2. Biochemical Response to Pollutant Exposure

#### 2.2.1. Pigment Content: Chlorophyll a and b, Carotenoids

The combined treatment with particulate matter (PM) and exposure to the elevated O_3_ and CO_2_ concentrations (PM + O_3_ + CO_2_) decreased or did not change the contents of chlorophyll a (chl a), chlorophyll b (chl b), and carotenoid in needles of Scots pine and Norway spruce seedlings and in leaves of small-leaved lime and Norway maple seedlings (Table 1). However, the content of chl a and chl b in silver birch leaves increased after the combined treatment. Significantly lower contents of chl a and chl b were found for Norway spruce and small-leaved lime, and the lower carotenoid content was found for Norway spruce compared to untreated samples. The carotenoid content in Scots pine, silver birch, small-leaved lime, and Norway maple seedlings did not respond to the combined PM + O_3_ + CO_2_ treatment.

Exposure to elevated combined O_3_ and CO_2_ concentrations without PM caused lower contents of chl a and chl b in Scots pine, Norway spruce, small-leaved lime, and Norway maple seedlings (Table 1). The largest decrease in chl a content was found in Norway spruce, small-leaved lime, and Norway maple. For the contents of chl b, the largest 21–30% decrease was found for small-leaved lime and Norway maple. The carotenoid content in small-leaved lime leaves increased by 8% (Table 1). In contrast, significantly lower carotenoid contents were observed in Scots pine, silver birch, and Norway maple leaves, with no change detected in Norway spruce compared to the control seedlings. When seedlings were treated with PM, the contents of both chl a and chl b decreased in Norway spruce and small-leaved lime, while only chl b decreased in Scots pine. In contrast, silver birch and Norway maple showed 1.2 to 1.3 times higher contents of chl a and chl b. Carotenoids did not respond to PM treatment.

#### 2.2.2. Total Polyphenol Content

**Figure 3:**
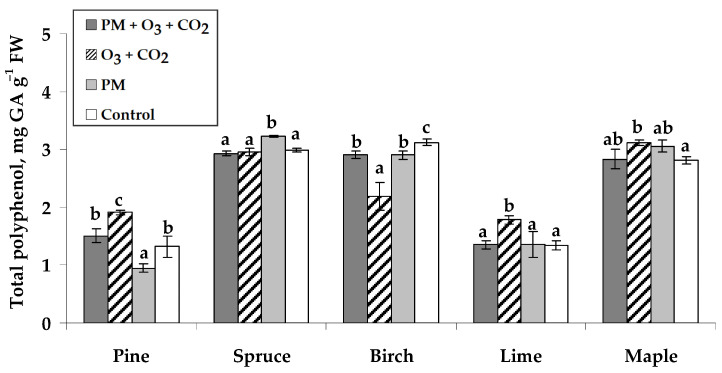
Total polyphenol content (±SE) in needles of Scots pine and Norway spruce seedlings and leaves of silver birch, small-leaved lime, and Norway maple seedlings. Experimental treatments: PM + O_3_ + CO_2_ is a treatment with particulate matter (PM) at elevated O_3_ and CO_2_; O_3_ + CO_2_—treatment without PM at elevated O_3_ and CO_2_; PM—treatment with PM at no elevated O_3_ and CO_2_; Control—no PM and no elevated O_3_ and CO_2_ (*n* = 9). Data significance was calculated using the Kruskal–Wallis test followed by Dunn–Bonferroni post hoc test for pairs (*p* ≤ 0.05). Different letters indicate statistically significant differences between treatments within each species.

The highest total polyphenol contents (TPC) in the control seedlings were found in Norway spruce needles (3.0 mg GA g^−1^), silver birch (3.1 mg GA g^−1^), and Norway maple (2.8 mg GA g^−1^) leaves (Figure 3). Seedling treatment with PM and elevated combined O_3_ and CO_2_ did not change the TPC in the needles of Scots pine and Norway spruce, as well as in the leaves of small-leaved lime and Norway maple. The elevated combined O_3_ and CO_2_ concentrations without PM treatment caused higher TPC in the needles of Scots pine (an increase of 45% was found) and in the leaves of small-leaved lime (33%) and Norway maple (11%). However, TPC decreased by 30% in silver birch leaves. The PM treatment responded with diverse effects on the TPC: from an 8% increase in Norway spruce needles, no change in the leaves of small-leaved lime and Norway maple, to a 28% decrease in Scots pine needles.

#### 2.2.3. Total Flavonoid Content

**Figure 4:**
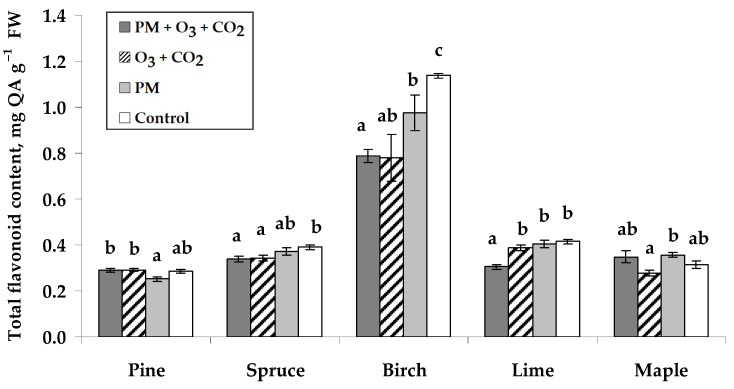
Total flavonoid content (±SE) in needles of Scots pine and Norway spruce seedlings and leaves of silver birch, small-leaved lime, and Norway maple seedlings. Experimental treatments: PM + O_3_ + CO_2_ is a treatment with particulate matter (PM) at elevated O_3_ and CO_2_; O_3_ + CO_2_—treatment without PM at elevated O_3_ and CO_2_; PM—treatment with PM at no elevated O_3_ and CO_2_; Control—no PM and no elevated O_3_ and CO_2_ (*n* = 9). Data significance was calculated using the Kruskal–Wallis test followed by Dunn–Bonferroni post hoc test for pairs (*p* ≤ 0.05). Different letters indicate statistically significant differences between treatments within each species.

Among the seedlings of different tree species, the highest total flavonoid contents (TFC) ranging between 0.78 and 1.14 mg QA g^−1^ of fresh weight were determined in silver birch leaves (Figure 4). Seedling treatment with PM and elevated combined O_3_ and CO_2_ resulted in a significant decrease in TFC in Norway spruce, silver birch, and small-leaved lime, respectively, by 13%, 31%, and 27%. The results showed a significant reduction in TFC after the elevated combined O_3_ and CO_2_ concentrations without PM treatment for Norway spruce and silver birch and after the PM treatment—for silver birch. There were no effects on TFC in the remaining species.

#### 2.2.4. Total Soluble Sugar Content

**Figure 5:**
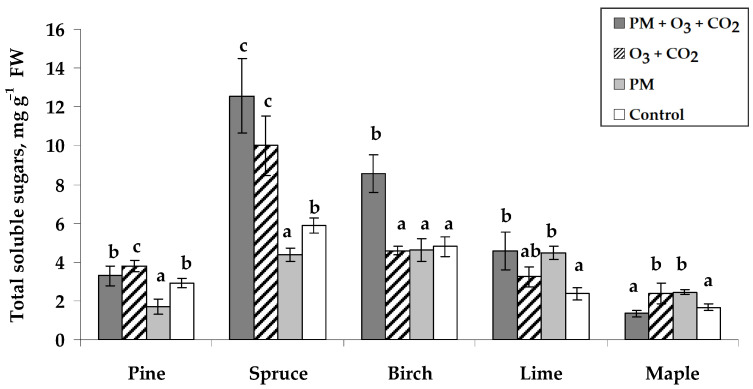
Total soluble sugar content (±SE) in needles of Scots pine and Norway spruce seedlings and leaves of silver birch, small-leaved lime, and Norway maple seedlings. Experimental treatments: PM + O_3_ + CO_2_ is a treatment with particulate matter (PM) at elevated O_3_ and CO_2_; O_3_ + CO_2_—treatment without PM at elevated O_3_ and CO_2_; PM—treatment with PM at no elevated O_3_ and CO_2_; Control—no PM and no elevated O_3_ and CO_2_ (*n* = 9). Data significance was calculated using the Kruskal–Wallis test followed by Dunn–Bonferroni post hoc test for pairs (*p* ≤ 0.05). Different letters indicate statistically significant differences between treatments within each species.

The highest total soluble sugar (TSS) content in the control seedlings was found in Norway spruce needles (5.9 mg g^−1^) and silver birch leaves (4.8 mg g^−1^) (Figure 5). Other tree species contained similar TSS content of 1.7–2.9 mg g^−1^. The content of TSS in seedlings of different tree species responded differently to the treatments. The treatment with PM applied together with elevated combined O_3_ and CO_2_ concentrations increased TSS in Norway spruce (113%), silver birch (78%), and small-leaved lime (93%). The seedling exposure to elevated combined O_3_ and CO_2_ concentrations without PM treatment significantly increased TSS in Scots pine, Norway spruce, and Norway maple seedlings (Figure 5). Following the PM treatment, TSS content decreased in Norway spruce by 26% and in Scots pine by 42%, while it increased by 89% in small-leaved lime and 47% in Norway maple.

**Figure 6:**
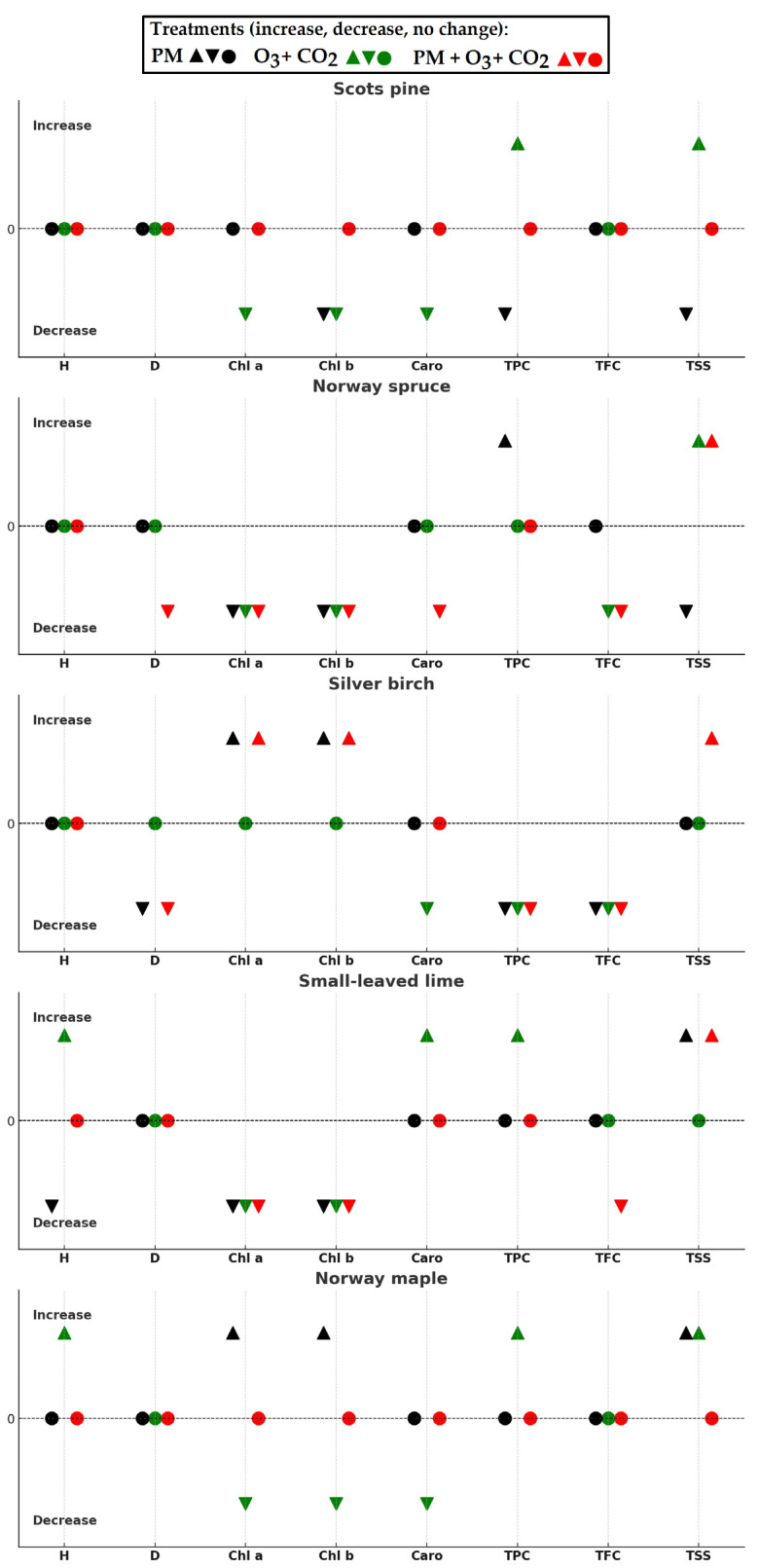
Overview of the effects observed in Scots pine, Norway spruce, silver birch, small-leaved lime, and Norway maple seedlings. Compared to the control (zero line), a significant increase in values is marked by upward-pointing (▲) black, green, and red triangles, a significant decrease—by downward-pointing (▼) black, green, and red triangles and black, green, and red circles (○) on the zero line indicates no change, where black is PM (particulate matter), green is O_3_ + CO_2_ (elevated O_3_ and CO_2_), and red is PM + O_3_ + CO_2_ (PM at elevated O_3_ and CO_2_). Note: On the x-axis, H represents the increment in stem height during the treatment period (twelve weeks), and D represents the increment in stem diameter at the root base per vegetation season. Biochemical compounds: Chl a is chlorophyll a; Chl b is chlorophyll b; Caro is the content of carotenoids; TPC is total polyphenol content; TFC is total flavonoid content; and TSS is total soluble sugars.

The effects of simulated pollution treatments on various growth and biochemical parameters obtained in the seedlings of different tree species were overviewed in Figure 6. In most cases, seedling growth parameters did not show significant differences after the seedlings had been exposed to varying conditions in the short term. The increased height was found for small-leaved lime and Norway maple after treatment with elevated combined O_3_ and CO_2_ without PM. The treatment with PM at elevated combined O_3_ and CO_2_ concentrations decreased the diameter of Norway spruce and silver birch, while the single PM treatment decreased the diameter of silver birch seedlings.

All treatments negatively influenced the concentrations of chl a and b in Norway spruce and small-leaved lime (Figure 6). However, positive effects were found for silver birch after treatment with PM at elevated combined O_3_ and CO_2_ and for silver birch and Norway maple after the single PM treatment. At elevated combined O_3_ and CO_2_ treatment, the PM was neutral to Scots pine and Norway maple seedlings, including all tested parameters—photosynthetic pigments (chl a and b, carotenoids), TPC, TFC, and TSS. Increased TPC was found in Scots pine, small-leaved lime, and Norway maple seedlings after exposure to the elevated combined O_3_ and CO_2_ concentrations without PM treatment. The TFC decreased in silver birch after all treatments, Norway spruce after exposure to elevated combined O_3_ and CO_2_ with and without PM, and small-leaved lime after exposure to elevated O_3_ and CO_2_ with PM. The treatment with PM at elevated combined O_3_ and CO_2_ caused an increase in TSS in Norway spruce, silver birch, and small-leaved lime, while elevated O_3_ and CO_2_ increased TSS in Scots pine, Norway spruce, and Norway maple. The single PM treatment decreased TSS in Scots pine and Norway spruce.

## 3. Discussion

### 3.1. Growth Response

Previous studies focused on the effect of trees and forests on air quality, most often causing the reducing air pollution and greenhouse gases in the environment [22,30,41,42]. Several studies have shown that trees growing in urban areas reduce air pollutants by intercepting and adsorbing PM through their foliage and removing gases via leaf stomata or plant surfaces [16,26,43,44,45,46]. However, PM accumulation in the leaves depends on various characteristics of leaves, such as surface roughness, epicuticular wax layer, and leaf shape and size [47]. Despite all these specific mechanisms, different species could be sensitive to environmental conditions, which could cause higher tree mortality or suggest selection preferences to urban planners [48].

The results of the present study indicated that elevated combined O_3_ and CO_2_ levels induced larger height increments in small-leaved lime and Norway maple seedlings (Figure 1). It is known that elevated CO_2_ concentrations cause more intensive plant growth followed by potentially higher biomass [49]. In contrast, O_3_ causes phytotoxic effects on vegetation [50]. More specifically, elevated CO_2_ enhances plant growth by improving photosynthetic carbon assimilation, though prolonged exposure may lead to photosynthetic down-regulation, especially under nutrient-limiting conditions. As noted by previous studies, O_3_ and CO_2_, acting together, generally negatively affect plant growth [51,52,53]. Elevated O_3_ concentrations diminish net CO_2_ assimilation, potentially causing significant losses in the carbon sink capacities of trees. The findings of this study did not reveal a negative impact from the combined influence of elevated O_3_ and CO_2_ levels. This could be attributed to either the short-term exposure duration or the evaluation of young tree seedlings, which could cause higher adaptability to varying environmental conditions.

We found that PM exposure without elevated combined O_3_ and CO_2_ had variable effects on different tree species. For example, PM treatment caused lower stem height growth in small-leaved lime seedlings (Figure 1). Species-specific responses were observed in stem diameter increments, i.e., all treatments resulted in a neutral effect or a decrease in diameter compared to the control (Figure 2). Norway spruce and silver birch seedlings experienced reduced diameter growth after the PM treatment at elevated combined O_3_ and CO_2_. The literature showed that atmospheric pollutants, including gas and PM, can have significant adverse impacts [54,55]. Recently, the impact of trees growing in urban areas on mitigating the adverse effects of PM in urban areas to remove air pollutants and improve air quality has been widely studied [56,57,58].

### 3.2. Biochemical Response

Assessing biochemical synthesis alterations in trees in response to air pollution stress provides insights into trees’ adaptive and physiological responses to environmental stressors. Our study demonstrates that the synthesis of biochemical compounds, including photosynthetic pigments, sugars, and secondary metabolites (TPC and TFC), is significantly impacted by pollution-induced stress. These findings highlight the sensitivity of biochemical compounds in trees to environmental pollutants. This study found that chl a and b concentrations decreased in Norway spruce and small-leaved lime after all treatments (Figure 6). In contrast, chl a and b concentrations increased in silver birch under the PM treatment at elevated combined O_3_ and CO_2_ and normal, unchanged conditions. TPC increased in Scots pine, small-leaved lime, and Norway maple at elevated combined O_3_ and CO_2_ concentrations. In contrast, under different treatments, TFC decreased in Norway spruce, silver birch, and small-leaved lime. TSS varied, increasing in several species with combined treatments and decreasing in Scots pine and Norway spruce with the single PM treatment. Previous studies found that the PM accumulation in foliage triggered chemical transformations, leading to alterations in the pH of leaf extracts. This shift in pH, whether an increase or decrease, depends on the specific compounds present in the PM. Elevated pH levels, commonly influenced by calcium or magnesium compounds, facilitate the entry of pollutants into leaf tissues, subsequently causing plasmolysis. Conversely, heightened acidity encourages the generation of radicals, which interact with cellular water, impacting the chlorophyll content in leaves [59]. Kováts et al. (2021) [60] have studied the effects of particulate pollution on several roadside plant species in Europe, taking chl a and b, carotenoids, and biomass as essential factors. In general, stress induced by air pollution triggers biochemical changes in plants, including a reduction in total chlorophyll content and an increase in ascorbic acid concentration [61]. When leaves absorb CO_2_ and PM, there is a potential for a decrease in the concentration of photosynthetic pigments such as chlorophylls and carotenoids. The study conducted with mung bean (*Vigna radiata* (L.) R. Wilczek) showed that plant leaves exposed to PM responded in a lower chl a/b ratio because of the shading effect from PM on leaves [62]. Consequently, this reduction can diminish photosynthesis and impact plant productivity [61,63].

Tree species are effective phytoremediators because they can remove pollutants, including gaseous contaminants such as O_3_ and CO_2_ [64]. This study concluded that silver birch, sycamore maple (*Acer pseudoplatanus* L.), and small-leaved lime were the most effective in this process. Łukowski et al. (2020) [65] discovered that silver birch was particularly effective in improving the quality of environments contaminated with PM. However, urban forest management should consider factors such as accelerated leaf fall, reduced productivity, and wood quality associated with this species.

Based on the evaluated stem growth and biochemical parameters in seedling foliage, specific general trends can be considered for trees growing in urban environments with enhanced PM and O_3_ levels and changing climates with higher CO_2_. Small-leaved lime and Norway maple seedlings showed height increments under elevated O_3_ and CO_2_ conditions.

A short-term study examining the early responses of young tree seedlings to PM, O_3_, and CO_2_ exposure in controlled conditions revealed species-specific response: Scots pine and Norway maple showed greater resilience to increased levels of these pollutants (no change in stem height, diameter, and TFC). Silver birch seedlings responded to higher chlorophyll content when exposed to single PM or PM combined with O_2_ and CO_2_, unlike other species. Silver birch is characterized as a species with high plasticity and stress protection mechanisms, which could have good acclimation capacity in a changing environment. High CO_2_ can potentially strengthen the oxidative stress tolerance of silver birch trees [66]. Increased CO_2_ levels stimulate photosynthesis and increase chlorophyll content, potentially mitigating the negative effects of PM and O_3_ in silver birch. This stress compensation may explain the increased chlorophyll levels observed in silver birch foliage under combined pollutant exposure [66,67]. Hence, our primary hypothesis that exposure to PM alone and the combined effects of elevated O_3_ and CO_2_ would result in species-specific growth and biochemical responses was largely confirmed. A greater impact of the PM at elevated O_3_ and CO_2_ was found for Norway spruce, silver birch, and small-leaved lime.

## 4. Materials and Methods

### 4.1. Experiment Design: Planting Material, Growing Conditions, and Treatments

The seedlings of five tree species—Scots pine (*Pinus sylvestris* L.), Norway spruce (*Picea abies* (L.) H.Karst.), silver birch (*Betula pendula* Roth), small-leaved lime (*Tilia cordata* Mill.), and Norway maple (*Acer platanoides* L.)—were selected for this study. These tree species are native to Lithuanian forests and are prioritized as the primary choices for planting in urban areas across the Baltic region, including Lithuania [68].

The planting material—high-quality seedlings—was purchased from Nemenčinė, Dubrava, Strošiūnai, and Kuršėnai tree nurseries, managed by the State Forest Enterprise (Vilnius, Lithuania) and Kołaki–Wietrzychowo tree nursery, managed by the Regional Directorate of State Forests (Białystok, Poland). According to available information, Scots pine seeds originated from Eastern Lithuania (Nemenčinė nursery), Norway spruce and silver birch seeds from Central Lithuania (Dubrava and Strošiūnai nurseries), and small-leaved lime seeds from Northern Lithuania (Kuršėnai nursery). Due to the unavailability of Norway maple seedlings in Lithuania, a tree nursery in Northeastern Poland (Kołaki–Wietrzychowo nursery), approximately 120 km from the Lithuanian border, was chosen, with seeds originating from that region. All seedlings grown in nurseries are mainly derived from forest reproductive material from seed orchards. The seedlings from tree nurseries are usually used for reforestation and afforestation, as well as for planting in urban areas in the region. The one-year-old tree seedlings of Scots pine and Norway spruce and two-year-old seedlings of silver birch, small-leaved lime, and Norway maple were replanted into individual 5 L plastic base-perforated pots containing substrate of neutralized peat (SuliFlor SF2 peat substrate, producer: Sulinkiai, Lithuania) in April 2022. The substrate contained a pH of 5.5–6.5 and optimum nutrition (total soluble N 210 mg L^−1^, P_2_O_5_ 240 mg L^−1^, and K_2_O 270 mg L^−1^). The seedlings were watered when necessary throughout this experiment. After one year of seedling growth, chlorine-free NPK liquid fertilizers (N 7.8%, NO_4_-N < 0.1%, NO_3_-N 2.0%, NO_2_-N 5.8%, P_2_O_5_ 2.3%, K_2_O 7.9%) with microelements Cu (0.002%) and Zn (0.005%) were applied to all tree seedlings before this experiment. The fertilizer solution was prepared and watered according to the dosage specified in the instructions (Baltic Agro, Lithuania). The potted seedlings were grown in an open field for one year before the simulation experiment began, making them two to three years old at the start of this experiment. We assumed that the young age of the seedlings—one year for coniferous species and two years for deciduous species at the time of purchase and two or three years, respectively, at the start of this experiment—would not represent a significant difference in seedling age.

A total of 84 seedlings from each tree species (Scots pine, Norway spruce, silver birch, small-leaved lime, and Norway maple) were divided into four groups of 21 seedlings, with each group assigned to one of the four treatments:

1. Seedlings treated with particulate matter (PM) and exposed to O_3_ levels of 180 ppb in combination with CO_2_ levels of 650 ppm from 9 a.m. to 9 p.m. (PM + O_3_ + CO_2_);
2. Seedlings without PM and exposed to an O_3_ level of 180 ppb in combination with a CO_2_ level of 650 ppm from 9 a.m. to 9 p.m. (O_3_ + CO_2_);
3. Seedlings with PM and exposed to O_3_ levels below 40–45 ppb in combination with CO_2_ levels below 400 ppm (representing unchanged air conditions) (PM);
4. Seedlings without PM were exposed to O_3_ levels below 40–45 ppb in combination with CO_2_ levels below 400 ppm, serving as the control group (Control).

For this experiment, the potted seedlings, with and without PM treatment, were placed in closed walk-in greenhouse chambers with regulated environmental conditions for twelve weeks, from mid-June to mid-September 2023. In the chambers, the seedlings were grown under natural light conditions; the air conditioning system maintained a temperature of 22 ± 2°C at day and 18 ± 2°C at night throughout this experiment. The air humidity was 60–75%. Temperature and air humidity were automatically adjusted.

For the PM pollution simulation, we used dry solid and dusty material obtained from a special multicyclone in the heating boiler located in Girionys, Kaunas district. This boiler is a part of the district heat production and supply company operating in the Kaunas region (Lithuania). This company primarily uses forest biomass for heating energy. We assumed that this pollution source, along with others, contributes to PM emissions in urban air, causing a significant environmental risk. To test this, we conducted experiments using a material with a known chemical composition (Table 2). The size of the PM particles was <10 µm (ISO 13322-1:2014; inverted microscope Nikon Eclipse Ts2, Japan with camera Lumenera Infinity 2 (Canada) 100× magnification). Tree seedlings received a single PM treatment before this experiment. Each seedling was exposed to 0.4 g of PM, which was manually applied as evenly as possible across the upper surfaces of the seedlings. All seedlings were treated with the same amount of PM under controlled indoor conditions, ensuring consistent environmental factors and minimal airflow disturbance. To evaluate early and sensitive changes, this study focused on evaluating biochemical responses in seedling foliage, which was directly affected by PM application and exposed under the selected environmental factors.

The O_3_ concentration of 180 ppb selected was four times higher than the ambient level typically recorded in rural and suburban regions of Lithuania [68]. Ozone from the air was generated using the ozone generator RMU16-6K (AZCO Industries Ltd., Canada). To control the O_3_ concentration, ozone transmitter E2638-03 (Evikon, Estonia) was used. A CO_2_ concentration of 650 ppm was selected following the RCP4.5 climate change scenario [69]. The CO_2_ was supplied to the air of the greenhouse chamber from compressed gas cylinders (Gaschema, Lithuania). The O_3_ and CO_2_ concentrations were controlled by the PC-based Environmental Control System (Computer software IGSS 9-13175). The combined treatments of PM + O_3_ + CO_2_ and O_3_ + CO_2_ were selected to represent urban environmental conditions. Due to limited technical capabilities during this experiment, (i) separate treatments for O_3_ and CO_2_ were not included, which limited the ability to evaluate their individual effects on tree seedlings; (ii) other environmental factors common in urban environments, such as fluctuating temperature, humidity, and other pollutants, were not evaluated, which limited the ecological applicability of the findings; and (iii) this study focused on specific tree species, limiting the generalizability of the findings to other species.

### 4.2. Measurements

#### 4.2.1. Seedling Stem Measurements

The height (cm) and diameter (mm) of seedlings were measured for all seedlings per treatment twice: at the beginning of this experiment (mid-June) and twelve weeks after the Scots pine, Norway spruce, silver birch, small-leaved lime, and Norway maple seedlings were grown in the chambers with simulated conditions (mid-September). The seedling growth data were analyzed by calculating the difference in growth indicators (shown as stem height and stem diameter increments in Figure 1 and Figure 2, respectively), using the values from mid-September minus those from mid-June.

#### 4.2.2. Biochemical Analyses

Needle samples of the Scots pine and Norway spruce seedlings and leaf samples of the silver birch, small-leaved lime, and Norway maple seedlings were collected from nine randomly selected seedlings per tree species from four treatments at the end of the 2023 vegetation season. Each sample was taken from the middle crown by pooling 10–20 needles and 4–6 leaves. All the samples of fully formed leaves and needles with no visible damage were collected on the same day. Only the leaves and needles from the current year were taken for analysis, meaning that they all grew for one growing season. Fresh needle/leaf samples were stored at −20 °C [70,71] for a short period, up to 1 month, until biochemical analyses were performed.

Quantification of amounts of photosynthetic pigments (chl a and b, carotenoids), total polyphenol content (TPC), total flavonoid content (TFC), and total soluble sugars (TSS) was performed spectrophotometrically using a SpectroStar Nano microplate reader (BMG Labtech, Offenburg, Germany) and 96-well microplates. A 0.1 g needle or leaf biomass was homogenized in a pestle and mortar and poured with 2 mL of 80% (*v*/*v* in water) ethanol, following the methodology by Čėsnienė et al. [71]. The samples were centrifuged for 30 min, 21,910× *g*, +4 °C, using a Hettich Universal 32R centrifuge (Andreas Hettich GmbH & Co. KG, Tuttlingen, Germany). The supernatant was then removed and used further.

For chl a, b, and carotenoids, analyses were performed with fresh extract in the dark to minimize component degradation by light. The absorption of the extract was measured at the wavelengths of 470 nm, 648 nm, and 664 nm. The concentration of chl a, b, and total carotenoids was calculated using the formulas produced by Lichtenthaler and Buschmann [72]:

C(chl a) = (13.36 × A664) − (5.19 × A648)(1)

C(chl b) = (27.43 × A648) − (8.12 × A664)(2)

C(carotenoids) = (1000 × A471 − 2.13 × C(chl a) − 97.64 × C(chl b))/(209)(3)

where A is the extract’s absorption at the respective wavelength; C(chl a), C(chl b), and C(carotenoids) are the concentrations of alpha and beta chlorophyll and total carotenoids in the extract (µg mL^−1^).

The photosynthetic pigment concentration in a gram of fresh leaf/needle mass was calculated according to the following formula:

C_x_ = ((C × V × W))/(M) (4)

where C_X_ is the concentration of pigments in fresh leaf/needle mass (µg g^−1^); C is the concentration of pigments in the extract (µg mL^−1^); V is the volume of crude extract (mL); W is the dilution of crude extract (units); M is the weight of extracted biomass (g).

Total polyphenol content (TPC) was determined using the Folin–Ciocalteu reagent using a modified methodology [73]. After one hour of incubation in the dark, sample absorption at a wavelength of 725 nm was measured. Gallic acid (>98%, Carl Roth GmbH + Co. KG, Karlsruhe, Germany) was used for the calibration curve. TPC was expressed as micrograms of gallic acid equivalent to one gram of fresh mass (mg g^−1^):

TPC (mg/g) = ((C × V))/(m)(5)

where C is the concentration obtained from the calibration curve (mg m L^−1^); V is the extract volume (mL); m is the weight of fresh biomass extracted (g).

Total flavonoid content (TFC) was estimated by forming a flavonoid–Al(III) complex [74]. Sample absorption at a wavelength of 415 nm was measured. Quercetin (>98%, Cayman Chemical Company, Ann Arbor, MI, USA) was used for the calibration curve. TFC was expressed as micrograms of the quercetin equivalent in one gram of fresh biomass (mg g^−1^) (Formula (5)).

Total soluble sugars (TSS) were determined using the methodology by Leyva et al. [75]. The supernatant was mixed with 0.1% anthrone reagent (CarlRoth, Karlsruhe, Germany) and heated at 90 °C for one hour (Agro-LAB Termostating TFC 200, Venice, Italy). The absorbance of the cooled samples was measured at a wavelength of 620 nm. Glucose was used to create the calibration curve, and the amount of soluble sugars was expressed as glucose equivalents (mg) per gram of raw tissue based on dilution and sample weight. The calibration curve. TSS was expressed as micrograms of the quercetin equivalent in one gram of fresh biomass (mg g^−1^) (Formula (5)).

### 4.3. Statistical Analysis

Lilliefors and Kolmogorov–Smirnov tests checked the normality of the variables. A non-parametric ANOVA Kruskal–Wallis analysis was used to ascertain the significant differences in growth and biochemical parameters between the treatments. This test was used instead of a standard one-way ANOVA as the data were non-normally distributed. To identify the significantly different means, the post hoc analyses were performed using the Dunn–Bonferroni procedure. Throughout this study, the means are presented with the standard error of the mean (±SE). Statistical analyses were conducted using Statistica 12.0 (StatSoft. Inc. 2007, Tulsa, OK, USA) software, and a level of significance of *p* ≤ 0.05 was chosen in all cases.

## 5. Conclusions

The growth and biochemical parameters of Scots pine (*Pinus sylvestris* L.), Norway spruce (*Picea abies* (L.) H.Karst.), silver birch (*Betula pendula* Roth), small-leaved lime (*Tilia cordata* Mill.), and Norway maple (*Acer platanoides* L.) showed an early response to particulate matter (PM) when exposed to elevated levels of combined O_3_ and CO_2_. Considering the technical limitations of this experiment, it was only possible to evaluate the combined effects of CO_2_ and O_3_ simultaneously rather than their individual effects. This study found that Scots pine and Norway maple were the most neutral species under the combined PM, O_3_, and CO_2_ treatments, showing no parameter changes. The treatment with a single PM gave a relatively diverse response by these species. The reaction of the chlorophyll parameter to PM exposure with or without elevated combined O_3_ and CO_2_ concentrations showed the following order of species: silver birch (increase in chl a and b)—Norway maple (neutral to increase)—Scots pine (neutral to decrease)—Norway spruce and small-leaved lime (decrease in chl a and b). Regarding the total polyphenol content (TPC), an active stress response was found in Scots pine, small-leaved lime, and Norway maple under increased combined O_3_ and CO_2_ and Norway spruce under PM treatments. While short-term exposure limits definitive conclusions, this study’s findings demonstrated that Scots pine and Norway maple showed higher resistance to increased PM and combined CO_2_ and O_3_ levels, as growth parameters did not change significantly. Silver birch seedlings showed an adaptive response by increasing chlorophyll content under pollutant exposure, indicating a higher capacity to adapt to urban pollutant stress. Even with these study conclusions, different variations in tree responses are likely to occur over a longer period as this experiment continues.

## Figures and Tables

**Table 1:** Mean chlorophyll a (chl a), chlorophyll b (chl b), and carotenoid content in needles of Scots pine and Norway spruce seedlings, as well as in leaves of silver birch, small-leaved lime, and Norway maple seedlings (*n* = 9). Data significance was calculated using the Kruskal–Wallis test followed by Dunn–Bonferroni post hoc test for pairs (*p* ≤ 0.05). Different letters in columns indicate statistically significant differences between treatments within each species.

Treatment *	Photosynthetic Pigment Content (µg g^−1^) ± SE		
	Chl a	Chl b	Carotenoid
	Scots pine seedlings		
PM + O_3_ + CO_2_	343.8 ± 10.6 ^ab^	290.1 ± 7.8 ^b^	14.6 ± 0.2 ^b^
O_3_ + CO_2_	325.1 ± 5.5 ^a^	262.2 ± 3.9 ^a^	13.9 ± 0.3 ^a^
PM	344.0 ± 23.8 ^ab^	268.1 ± 16.4 ^a^	14.4 ± 0.5 ^ab^
Control	366.3 ± 10.8 ^b^	283.6 ± 1.3 ^b^	14.8 ± 0.1 ^b^
	Norway spruce seedlings		
PM + O_3_ + CO_2_	428.9 ± 5.5 ^a^	339.8 ± 7.1 ^a^	15.4 ± 0.6 ^a^
O_3_ + CO_2_	459.6 ± 13.9 ^b^	349.2 ± 4.1 ^a^	17.3 ± 0.6 ^b^
PM	532.7 ± 5.4 ^c^	393.9 ± 4.1 ^b^	17.1 ± 0.2 ^b^
Control	615.9 ± 18.3 ^d^	427.8 ± 12.6 ^c^	17.3 ± 0.3 ^b^
	Silver birch seedlings		
PM + O_3_ + CO_2_	385.4 ± 27.8 ^b^	271.7 ± 17.3 ^b^	20.0 ± 0.5 ^ab^
O_3_ + CO_2_	308.0 ± 8.8 ^a^	226.8 ± 4.4 ^a^	19.6 ± 0.7 ^a^
PM	413.5 ± 36.4 ^b^	292.4 ± 25.3 ^b^	21.0 ± 0.6 ^ab^
Control	306.3 ± 14.8 ^a^	228.2 ± 7.3 ^a^	21.0 ± 0.2 ^b^
	Small-leaved lime seedlings		
PM + O_3_ + CO_2_	411.9 ± 9.7 ^a^	296.7 ± 4.9 ^a^	28.6 ± 1.6 ^ab^
O_3_ + CO_2_	595.5 ± 33.5 ^b^	398.2 ± 20.3 ^b^	29.7 ± 0.8 ^b^
PM	748.6 ± 36.0 ^c^	459.2 ± 20.0 ^c^	28.3 ± 0.7 ^ab^
Control	848.7 ± 24.8 ^d^	503.3 ± 10.6 ^d^	27.5 ± 1.1 ^a^
	Norway maple seedlings		
PM + O_3_ + CO_2_	567.5 ± 26.5 ^b^	394.8 ± 27.0 ^b^	25.2 ± 1.9 ^ab^
O_3_ + CO_2_	445.7 ± 26.4 ^a^	295.8 ± 17.9 ^a^	22.9 ± 1.0 ^a^
PM	815.3 ± 19.3 ^c^	520.2 ± 11.3 ^c^	24.2 ± 0.7 ^ab^
Control	619.6 ± 32.7 ^b^	421.6 ± 19.4 ^b^	25.1 ± 0.3 ^b^

* Note: PM + O_3_ + CO_2_ is the treatment with particulate matter (PM) at elevated O_3_ and CO_2_; O_3_ + CO_2_ is the treatment without PM at elevated O_3_ and CO_2_; PM is the treatment with PM at no elevated O_3_ and CO_2_; Control indicates no PM, no elevated O_3_, and CO_2_.

**Table 2:** The characteristics of the dry material used as an alternative to particulate matter (PM).

Parameter	Value	Analysis Method
pH	12	ISO 10390:2021
Organic carbon (C, %)	3.03	LST EN 15936:2022
Phosphorus (P, mg kg^−1^)	14,352	LST EN 13657:2003, LST EN ISO 6878:2004
Potassium (K, mg kg^−1^)	15,000	LST EN 13657:2003, ISO 9964-3:1993
Calcium (Ca, mg kg^−1^)	237,250	LST EN 13657:2003, LST EN ISO 7980:2000
Magnesium (Mg, mg kg^−1^)	30,083	
Cadmium (Cd, mg kg^−1^)	13.5	LST EN 13657:2003, LST EN ISO 11885:2009
Arsenic (As, mg kg^−1^)	3.00	
Nickel (Ni, mg kg^−1^)	19.2	
Lead (Pb, mg kg^−1^)	98.0	
Boron (B, mg kg^−1^)	457	
Vanadium (V, mg kg^−1^)	9.3	
Chromium (Cr, mg kg^−1^)	58.8	
Copper (Cu, mg kg^−1^)	135	
Zink (Zn, mg kg^−1^)	2947	
Mercury (Hg)	0.143	LST EN 13657:2003, LST EN ISO 12846:2012
Benzo(a)pyrene	<0.5	LST EN 17503:2022

## Data Availability

The original contributions presented in this study are included in this article; further inquiries can be directed to the corresponding author.
